# Overcoming Multidrug‐Resistant MRSA Using Conventional Aminoglycoside Antibiotics

**DOI:** 10.1002/advs.201902070

**Published:** 2020-03-14

**Authors:** Lei Tan, Ziao Zhou, Xiangmei Liu, Jun Li, Yufeng Zheng, Zhenduo Cui, Xianjin Yang, Yanqin Liang, Zhaoyang Li, Xiaobo Feng, Shengli Zhu, Kelvin Wai Kwok Yeung, Cao Yang, Xianbao Wang, Shuilin Wu

**Affiliations:** ^1^ Hubei Key Laboratory of Polymer Materials Ministry‐of‐Education Key Laboratory for the Green Preparation and Application of Functional Materials School of Materials Science & Engineering Hubei University Wuhan 430062 China; ^2^ School of Materials Science & Engineering The Key Laboratory of Advanced Ceramics and Machining Technology by the Ministry of Education of China Tianjin University Tianjin 300072 China; ^3^ State Key Laboratory for Turbulence and Complex System and Department of Materials Science and Engineering College of Engineering Peking University Beijing 100871 China; ^4^ Department of Orthopaedics Union Hospital Tongji Medical College Huazhong University of Science and Technology Wuhan 430022 China; ^5^ Department of Orthopaedics & Traumatology Li Ka Shing Faculty of Medicine The University of Hong Kong Pokfulam Hong Kong China

**Keywords:** aminoglycoside antibiotics, antibacterial effect, MRSA, photothermal treatment, red phosphorus nanoparticles

## Abstract

Global multidrug‐resistant (MDR) bacteria are spreading rapidly and causing a great threat to human health due to the abuse of antibiotics. Determining how to resensitize MDR bacteria to conventional inefficient antibiotics is of extreme urgency. Here, a low‐temperature photothermal treatment (PTT, 45 °C) is utilized with red phosphorus nanoparticles to resensitize methicillin‐resistant *Staphylococcus aureus* (MRSA) to conventional aminoglycoside antibiotics. The antibacterial mechanism is studied by the proteomic technique and molecular dynamics (MD) simulation, which proves that the aminoglycoside antibiotics against MRSA can be selectively potentiated by low‐temperature PTT. The catalytic activity of 2‐aminoglycoside phosphotransferase (APH (2″))—a modifying enzyme—is demonstrated to be obviously inhibited via detecting the consumption of adenosine triphosphate (ATP) in the catalytic reaction. It is also found that the active site of aspartic acid (ASP) residues in APH (2″) is thermally unstable from the results of molecular dynamics simulation. Its catalytic ability is inhibited by preventing the deprotonating procedure for the target —OH of gentamycin. The combined therapy also exhibits great biocompatibility and successfully treats MRSA infections in vivo. This low‐temperature PTT strategy has the potential to be an exogenous‐modifying enzyme inhibitor for the treatment of MDR bacterial infection.

## Graphical abstract

Resensitizing multidrug‐resistant bacteria to conventional inefficient antibiotics is of extreme urgency. Low‐temperature photothermal treatment as an exogenous modifying enzyme inhibitor can resensitize methicillin‐resistant *Staphylococcus aureus* (MRSA) to conventional aminoglycoside antibiotics selectively. The active site of aspartic acid residues in 2‐aminoglycoside phosphotransferase is found to be thermally unstable, which prevents the deprotonating procedure to the target —OH of gentamycin and inhibits the catalytic ability of the enzyme.

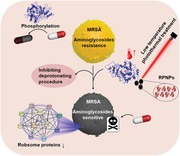

The escalating tide of antibiotic‐resistant bacterial infections introduces a tremendous economic burden and seriously threatens human health care worldwide. The World Health Organization reported that over 2 million people are infected by antibiotic‐resistant pathogens, with 2300 dying every year.^1^ As one of the three main threats to human health, the morbidity and mortality that are caused by antibiotic‐resistant pathogens has been predicted to exceed the threat of cancer in the near future.^2^ Since conventional antibiotics gradually become inefficient and face the problem of being phased out, the discovery of new effective antibiotics is urgently needed. Many new synthetic antibacterial drugs, such as teixobactin,^3^ retinoids,^4^ or antimicrobial peptides,^5^ exhibited efficient antibacterial performance against antibiotic‐resistant pathogens, which shows great potential for infection therapeutics. Generally, a new antibiotic needs 10 or more years before it becomes available in clinical practice, which will cost much manpower and money. However, the time it takes for a pathogen to develop resistance is less than two weeks.^6^ To address the urgent need for the treatment of antibiotic‐resistant bacterial infections, we still depend on conventional antibiotics. The utility of these antibiotics is eroded by various drug‐resistant mechanisms, which can be classified into three categories: 1) generating a modifying enzyme, 2) altering the targets of antibiotics, 3) or changing the bacterial membrane permeability and efflux.^7^ Inhibiting these resistant mechanisms is the most effective strategy to extend the life of conventional antibiotics. Various adjuvants, such as clavulanic acid (β‐lactamase inhibitor), celecoxib (efflux pump inhibitor), and quercetin (aminoglycoside‐modifying enzyme inhibitor) were utilized to overcome resistance.^8^ Although scientists have paid much attention to the improvement of conventional antibiotics through combining them with drugs of resistant inhibitors or using dual antibiotics, their toxicity, pharmacokinetic differences, and promoted evolution of drug resistance still limit their application.^7^

Therefore, we must find a new strategy to resensitize antibiotic‐resistant pathogens to conventional antibiotics without side effects, thus prolonging the life of old drugs. Unlike utilizing traditional antibacterial drugs, inhibiting the resistant mechanism through an exogenous strategy, such as low‐temperature photothermal therapies (PTT), has never been reported. PTT for cancer or bacterial infection have been studied by researchers.^9^ Although antibiotics such as daptomycin and vancomycin are combined with a photothermal effect using near‐infrared (NIR) light to treat methicillin‐resistant *Staphylococcus aureus* (MRSA) infection, these antibiotics are not inefficient.^10^ Moreover, to realize a lethal photothermal effect, the temperatures that were used were still above 50 °C, which may have damaged the healthy tissue.[qv: 9g] Thus, we are wondering if using a mild temperature below 50 °C can inhibit the resistant mechanism of MRSA without a lethal effect and resensitize it to conventional antibiotics.

In the application of PTT, a degradable and biocompatible PTT agent is necessary. The black phosphorus nanoparticles (BPs) was previously reported as a promising and safe PTT agent^11^ because phosphorus is one of the vital elements in the human body and BPs can be degraded into biocompatible phosphate and hydrogen phosphate ions.^12^ Compared with BPs, red phosphorus (RP) with the same degradation products is also nontoxic.^12^ Here, the red phosphorus nanoparticles (RPNPs) that are prepared by the chemical vapor deposition (CVD) method are used as a new photothermal agent for the first time, which possessed great biocompatibility and a photothermal conversion property. Then, a low temperature PTT (45 °C) based on RPNPs is combined with four different types of inefficient antibiotics, including penicillin (Pen), roxithromycin (Rox), tetracycline (Tet), and four kinds of aminoglycosides (gentamycin sulfate C1 (Gen), kanamycin sulfate (Kan), amikacin disulfate (Ami), and sisomicin sulfate (Sis)) to study their antibacterial performance against multidrug‐resistant MRSA in vitro and in vivo infection models. We aim to study which kinds of antibiotics can be potentiated in the presence of PTT and to investigate the mechanism of inhibiting antibiotics resistance by using a proteomic technique and molecular dynamics (MD) simulation.

To prepare RPNPs, ZnO particles were first deposited by RP by using the CVD method. Then, the ZnO/RPNPs were treated by hydrochloric acid and ultrasonication to obtain RPNPs (**Figure** **1**a). As shown in Figure 1b, the average size of RPNPs was approximately 120 nm, and the P element in the nanoparticles was confirmed by an inserted energy‐dispersive spectrometry (EDS) spectrum. The parallel lattice fringes of (400) facets with a d‐spacing of 2.78 Å and (001) facet with a d‐spacing of 5.80 Å proved that the fibrous phase phosphorus existed in the RPNPs (Figure 1c).[qv: 9g] Typical Raman peaks from 300 and 500 cm^−1^ of both RP and RPNPs proved that these nanoparticles were red phosphorus (Figure 1d). From Figure 1e, we can observe that the dark black color of RPNPs compared with the dark red color of RP and the absorbance of RPNPs dispersion was much stronger than RP covering the NIR region. The strong absorbance of photothermal agents will benefit their photothermal‐conversion ability. Next, the photothermal heating curves of RPNPs and RP were measured by an infrared thermal camera under the irradiation of an 808 nm laser (1 W cm^−2^). The temperatures of the RPNPs with different concentrations of 100, 200, and 400 µg mL^−1^ increased to 56.5, 64.2, and 74.5 °C after 6 min, respectively, and were kept stable over time. In contrast, the temperature of RP only increased from 28.1 to 31.8 °C for RP after 10 min irradiation, which indicates that the photothermal‐conversion ability increased significantly from RP to RPNPs (Figure 1f). The photothermal heating curve exhibited higher repeatability during recycling, which suggests that RPNPs is a durable photothermal agent (Figure 1g). The photothermal‐conversion efficiency (η) of the RPNPs was calculated to be 35.5% (Figure 1h), which was much higher than the Au nanoshells (13%),^13^ Au nanorods (21%),^14^ and Cu_9_S_5_ nanocrystals (25.7%).^15^

**Figure 1:**
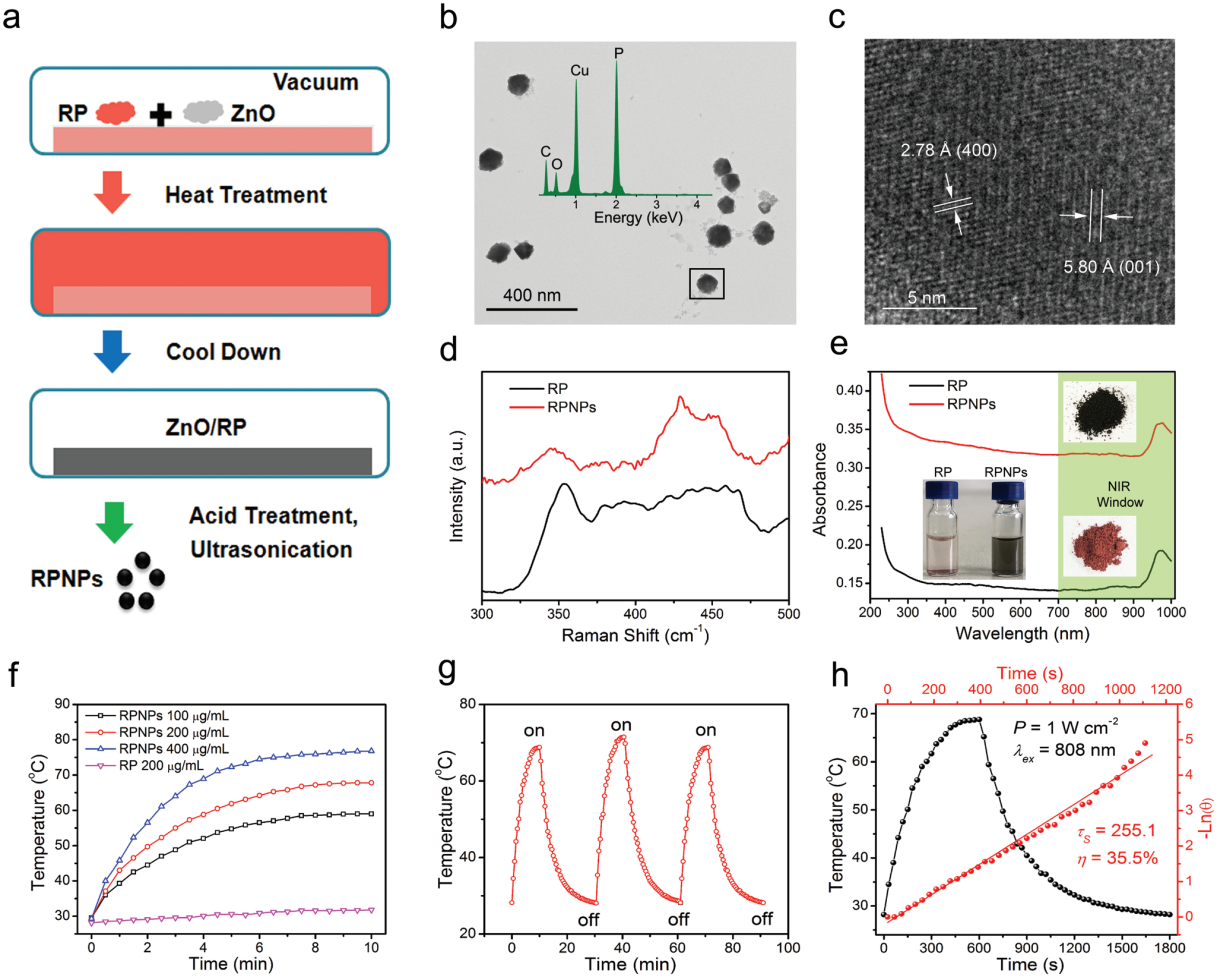
a) Synthetic routine of RPNPs. b) Transmission electron microscopy (TEM) and EDS image of RPNPs. c) High‐resolution TEM image of RPNPs. d) Raman characterization of RP and RPNPs. e) Absorption of RP and RPNPs aqueous dispersion and their corresponding images. f) Temperature changes of RPNPs (100, 200, and 400 µg mL^−1^) and RP (200 µg mL^−1^) under 808 nm laser (1 W cm^−2^). g) Temperature changes of RPNPs (200 µg mL^−1^) for three laser on/off cycles under 808 nm laser (1 W cm^−2^). h) Calculation of the photothermal‐conversion efficiency (η) of RPNPs under a 808 nm laser.

Because of the MDR properties of MRSA, many proteins participate in resistance processes to invalidate antibiotics, and reducing the activity of these proteins may weaken their resistance behaviors. We hypothesized that a low‐temperature PTT treatment (45 °C) may inhibit the resistance process to make some antibiotics efficient again. Pen, Rox, Tet, and aminoglycoside antibiotics including Gen, Kan, Ami, and Sis were combined with the PTT treatment. From the bacterial growth curves (**Figure** **2**a), we found that the individual treatment of Gen (1 × MIC) or RPNPs (all of the “RPNPs” in the following text were under the following condition: 45 °C for 30 min) showed no obvious difference compared with the control group (no treatment) over 8 h, which suggests that this concentration of Gen or temperature was too low to influence the growth of MRSA. However, after combination, the growth speed of MRSA was much slower than that of others, and the number of colony forming units (CFU) per mL was reduced by 29‐fold compared with the control, which indicates that the inefficient Gen (1 × MIC) could become efficient again with the help of PTT. Next, the concentration of Gen was elevated, as shown in Figure 2b, and the growth of MRSA was kept static until 8 h due to the increased content of Gen (4 × MIC). With the addition of PTT, the Gen (4 × MIC) resulted in an approximate 53‐fold decrease in the MRSA burden compared with Gen (4 × MIC) alone. Although Gen increased to 16 × MIC or 32 × MIC, the antibacterial performance was still inferior to the group of RPNPs‐Gen (4 × MIC), which suggests that the combination of Gen and PTT could significantly decrease MRSA abundance, which would avoid side effects without having to use high concentrations of antibiotics in antibiotic treatments. Given that the Gen can be potentiated by PTT, we investigated whether other aminoglycoside antibiotics, including Kan (1 × MIC), Ami (1 × MIC), and Sis (0.5 × MIC), could also be potentiated. As expected, the antibacterial performance of these three kinds of aminoglycoside antibiotics were all enhanced obviously compared with antibiotic treatment alone in the presence of PTT. To investigate if this potentiation was applied to other types of inefficient antibiotics, Rox (0.25 × MIC), Pen (0.5 × MIC), and Tet (0.5 × MIC) were also combined with PTT. However, Pen and Tet showed no obvious potentiation, and the CFU per mL of MRSA in the Rox group only decreased within twofold (Figure 2c). These results imply that this PTT treatment might specifically disable MRSA aminoglycosides antibiotic resistance and resulted in potentiation in vitro. Drug‐resistant mechanisms are mainly classified into three categories: 1) generating a modifying enzyme, 2) altering the targets of the antibiotic, 3) or changing the bacterial membrane permeability and efflux.^7^ Since the antibiotic targets formed before applying PTT, this PTT might influence the other two drug‐resistant mechanisms. Therefore, we next investigated the bacterial membrane permeability change of MRSA after treatments. Subsequently, ANS (8‐anilinonapthalene‐1‐sulfonic acid) was used to measure the membrane permeability of MRSA due to its increased fluorescence when combined with hydrophobic membrane regions. From Figure 2d, an obvious increased fluorescence caused by 1% TritonX‐100 (positive control) was observed. However, there is no significant difference between each group after treatments, which indicates that the single or combinatory treatment did not change the bacterial membrane permeability of MRSA. Moreover, no more protein leakage was observed in the other three groups compared with that of the control (no treatment), which further proved that the membrane permeability was unchanged (Figure 2e). Since the drug‐resistant mechanism of Tet and Rox is efflux,^16^ and Pen can be combined with PBP2a encoded by MRSA,^17^ low‐temperature PTT may be unable to inhibit these procedures. From the above, the main reason for the resensitization of MRSA to aminoglycoside antibiotics was that the modifying enzyme might be inhibited by low‐temperature PTT. We also demonstrated that the MRSA biofilm could be eradicated successfully (Figures S1–S4, Supporting Information) due to the potentiation effect of the PTT and the small size of the RPNPs.^18^

**Figure 2:**
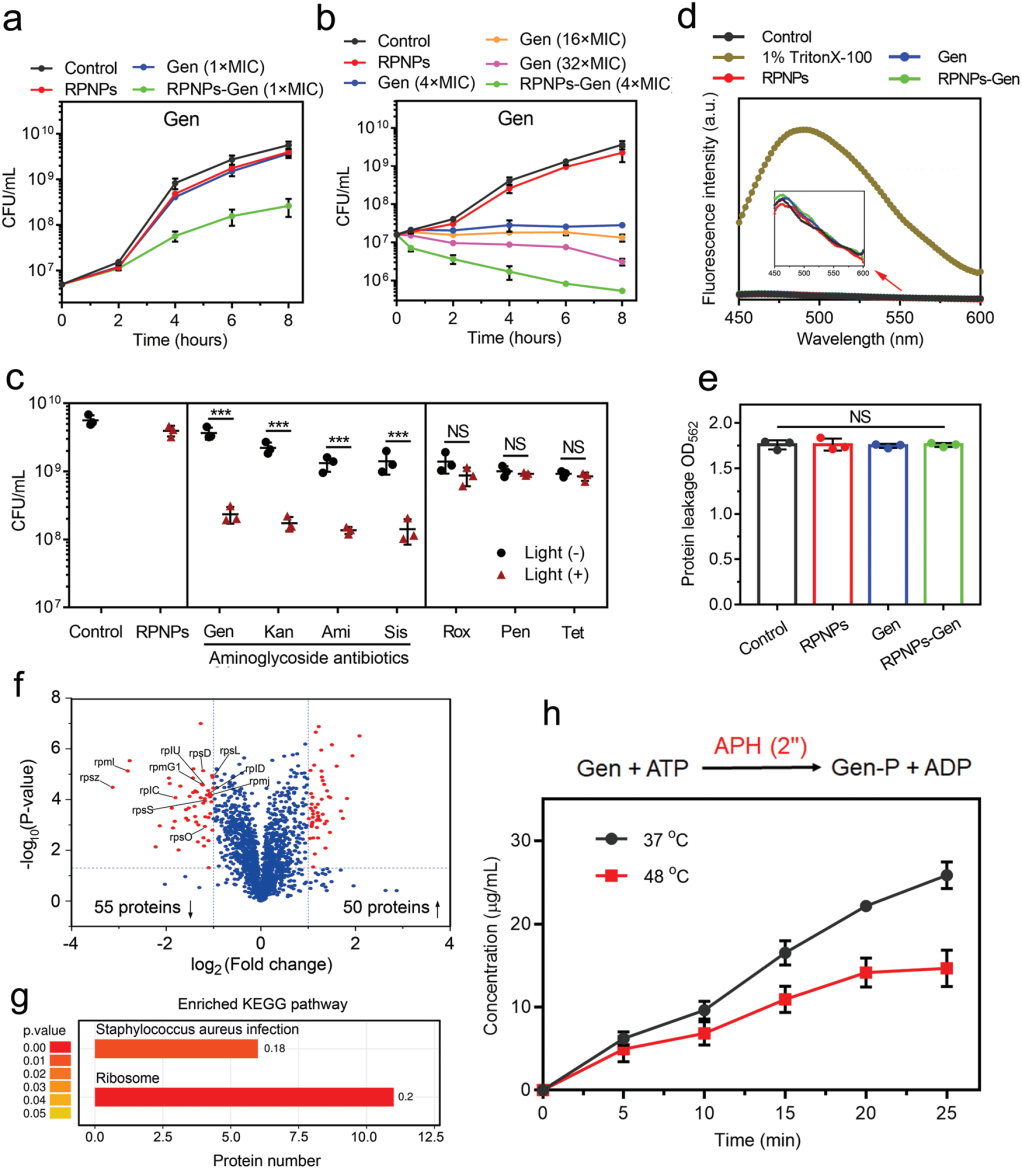
a) Bacterial growth curves of control (no treatment), Gen (1 × MIC), RPNPs, and RPNPs‐Gen (1 × MIC) groups for 8 h. The concentration of initial bacterial suspension is 5 × 10^6^ CFU per mL. b) Bacterial growth curves of the control (no treatment), Gen (4 × MIC), Gen (16 × MIC), Gen (32 × MIC), RPNPs, and RPNPs‐Gen groups (4 × MIC) for 8 h. The concentration of the initial bacterial suspension is 1.6 × 10^7^ CFU per mL. c) The antibacterial performance of Pen, Rox, Ter, and aminoglycoside antibiotics include Gen, Kan, Ami, and Sis combined with PTT after 8 h. *n* = 3 independent experiments per group, ***P < 0.001. d) MRSA membrane permeability determined by ANS uptake assay. e) Protein leakage from MRSA determined by a BCA kit after 30 min treatment. f) Volcano plot (the pointed proteins were attributed to ribosome proteins) show the upregulated and downregulated proteins of MRSA treated by Gen and RPNPs‐Gen (RPNPs‐Gen vs Gen, fold change >2, *P* < 0.05). g) Enriched KEGG pathway results show that the pathway of the *Staphylococcus aureus* infection and ribosome had significant change. h) Chemical reaction equation of phosphorylation. The release curve of phosphorylated Gen (Gen‐P) at different temperature was calculated from the consumption of ATP measured by ATP assay kit. *n* = 3 per group.

The iTRAQ‐based quantitative proteomic technique was performed to investigate the antibacterial mechanism of combination treatment and the reason why the low‐temperature PTT could selectively potentiate aminoglycoside antibiotics. Since the Gen had almost no inhibition on the growth of MRSA, we chose two groups of RPNPs‐Gen and Gen for this analysis to study the influence of the PTT effect on the drug efficacy. Among the identified 1916 proteins, after the combination of PTT treatment, 50 upregulated and 55 downregulated differential proteins were detected (RPNPs‐Gen vs Gen, fold change >2, *P* < 0.05) (Figure 2f; Figure S5, Supporting Information). As shown in Figure S6a (Supporting Information), the proteins that are related to cell killing and extracellular region were upregulated in the RPNPs‐Gen group due to the excretion of dead MRSA. In addition, the proteins related to structural molecule activity and the organelles, which belonged to the ribosome, were all downregulated. Kyoto Encyclopedia of Genes and Genomes (KEGG) pathway analysis was used to study the change of signaling pathway of MRSA after treatments. As shown in Figure 2g, we found that the enriched KEGG pathways of *Staphylococcus aureus* infection and ribosome had a significant change. The *Staphylococcus aureus* infection pathway involved some toxin factors released from the dead MRSA, which indicates that the group of RPNPs‐Gen exhibited more serious damage than Gen. The most frequently activated pathway was the ribosome pathway. There were 11 proteins that participated in this pathway were downregulated (indicated in Figure 2f), which suggested that the inefficient Gen became efficient again. This biological process was consistent with the antibacterial mechanism of Gen, which could bind with the ribosomal 30S subunit of bacteria to inhibit protein synthesis.^19^ The protein–protein interaction (PPI) result also showed that all of the 11 ribosomal proteins had a strong interaction and belonged to the ribosomal pathway (Figure S6b, Supporting Information). The above results proved that the aminoglycoside antibiotics against MRSA could be potentiated by PTT. To investigate which proteins resulted in this potentiation, from the identified 1916 proteins (Table S1, Supporting Information), two aminoglycoside‐modifying enzymes, including aminoglycoside nucleotidyltransferase (ANT(9)‐Ia, ‐1.1‐fold ∆) and 2‐aminoglycoside phosphotransferase (APH (2″), ‐1.1‐fold ∆), were found and showed a slight decrease when PTT was applied. In addition, the PBP2a (2.1‐fold ∆) and adenosine triphosphate (ATP)‐binding cassette domain‐containing protein (3.8‐fold ∆) with the efflux property were also detected and showed an obvious increase, which proved that Tet, Rox, and Pen could not be potentiated by PTT. Specially, the 2″‐hydroxy of Gen, Kan, Ami and Sis could be phosphorylated by APH (2″).^20^ To investigate whether the activity of APH (2″) was inhibited by the thermal treatment, the phosphorylation reactions of Gen were performed at 37 and 48 °C, respectively. The reason we chose 48 °C is that the antibacterial behavior of PTT at 45 °C when using nanoparticles showed no obvious difference in the treatment by water bath at 48 °C due to the localized heating effect of nanoparticles (Figure S7, Supporting Information).^21^ Since the ATP can be turned into adenosine diphosphate (ADP) during the phosphorylation procedure of Gen, the content change of ATP in this reaction was detected by an ATP Assay Kit (Beyotime) at 37 and 48 °C, respectively. From Figure 2h and Figure S8 (Supporting Information), the release curve of phosphorylated Gen (Gen‐P) at different temperature was calculated from the consumption of ATP measured by ATP assay kit. It can be observed that the generation of Gen‐P at 48 °C was obviously lower than at 37 °C, indicating the inhibition of APH (2″) activity under a higher temperature. Based on the results of Table S1 (Supporting Information) about the whole protein quantification and differential analysis from proteomic, only two aminoglycoside‐modifying enzymes including nucleotidyltransferase (ANT(9)‐Ia) and 2‐aminoglycoside phosphotransferase (APH (2″)) were detected (highlighted in Table S1), which related to the resistant mechanism of MRSA toward aminoglycoside antibiotics. From the molecular structure of Gen we chose, the Gen only can be modified by APH (2″). So, if the activity of APH (2″) was inhibited, the MRSA could be resensitized to Gen. We next investigated why the activity of APH (2″) was inhibited in the presence of PTT.

To better understand the inactivation mechanism of 2‐aminoglycoside phosphotransferase APH (2″) by thermal treatment, an MD simulation was performed to analyze the conformation change of APH (2″) and to examine the interaction between APH (2″) and Gen at 37 and 48 °C (100 ns), respectively. The root mean square deviation (RMSD) of the protein backbone at different temperatures was calculated to investigate the conformational stability of structures. As shown in **Figure** **3**a, the RMSD value of protein at 37 °C was approaching equilibrium after 30 ns, which indicates that the conformation became stabile at this temperature. In the case of 48 °C, the backbone of the protein showed higher fluctuation with time and was unstable compared with the structure at 37 °C. The root mean square fluctuation (RMSF) reflected the flexibility of the amino acid residue. In these 479 residues, except for the loop residues (56–58, 174–180, 342–344, 436–438, and 467–469) with high fluctuation between two different temperatures, the residues in the regions 247–256, 315—327, and 390–406 also exhibited obvious fluctuation, which might have an influence on the activity sites of APH (2″) (Figure 3b). In addition, the gyration radius of APH (2″) at 48 °C was obviously increased compared with that at 37 °C, which suggests that the higher temperature could result in a looser conformation and reduce the stability of the protein or affect its catalytic activity (Figure 3c). The looser structure at 48 °C was also observed in the protein conformation image (Figure 3d). Next, the molecular docking simulations of APH (2″) with Gen and ATP were performed to investigate their interaction change at 37 and 48 °C, respectively. As shown in Figure 3d, we can observe that the residues including TYR79, ASN106, SER109, ILE112, TYR223, SER318, GLU319, ASP393, ILE400, and TYR408 interacted with Gen at 37 °C, while the residues of ILE224, ASN232, LYS233, LYS241, SER291, GLU292, GLU319, THR321, ASP323, and ASN327 interacted with Gen at 48 °C, which suggests that the increase of the temperature changed the interaction sites of APH (2″) and Gen. Among these residues, the ASP393 and ASP323 were more thermally unstable than the other residues that were referred to the RMSF result. Since ASP (aspartic acid) residues play an important role in the catalysis reaction of protein kinases, including APH, which can participate in deprotonating the target —OH of Gen and enhancing its nucleophilicity,^22^ the catalytic ability of ASP residue might be inhibited by thermal treatment. In addition, after thermal treatment, the increased distance from the oxygen of ASP residue (indicated by red circle) to the 2″‐hydroxy of Gen (indicated by a black circle) and the newly formed hydrogen bond with ILE‐224 residues further weakened the catalytic process. Therefore, it might be difficult for the hydrogen bond between the ASP residue and 2″‐hydroxy to form for the activate phosphorylation reaction (Figure 3e). Moreover, the docking energy between APH (2″) and Gen was changed from −12.97 kcal mol^−1^ (37 °C) to −8.04 kcal mol^−1^ (48 °C), which indicates that their binding capacity was weaker at a higher temperature and was not conducive to the catalysis (Figure 3f). In the actual PTT treatment, the inactivation effect of APH (2″) might be amplified under 30 min heating compared with the MD simulation time of 100 ns.

**Figure 3:**
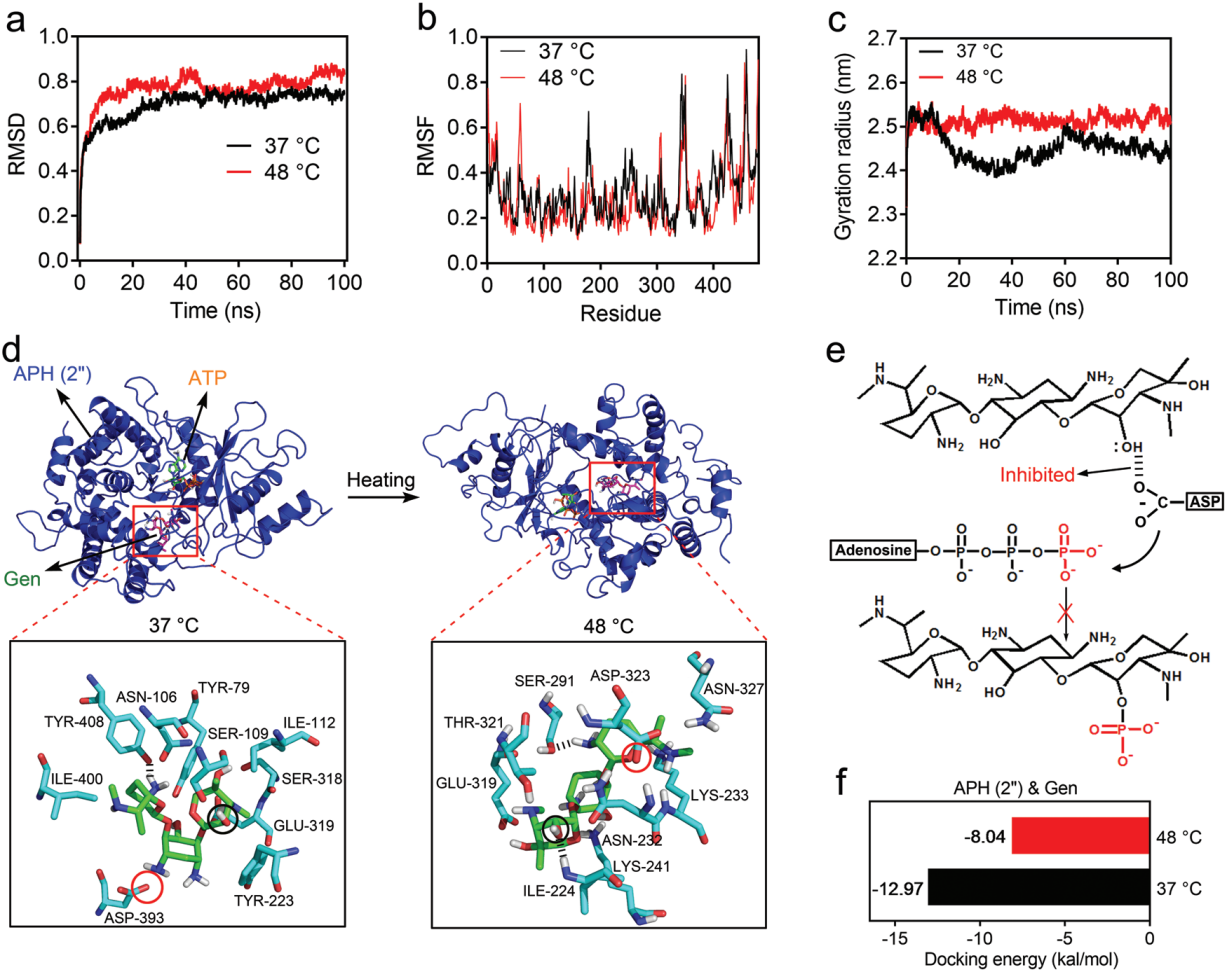
a) RMSD, b) RMSF, and c) gyration radius of APH (2″) at 37 and 48 °C, respectively. d) Molecular docking simulations of APH (2″) with Gen and ATP at 37 and 48 °C (100 ns), respectively. e) Phosphorylation reaction of Gen. f) Docking energy between APH (2″) and Gen at 37 and 48 °C, respectively.

Next, the biocompatibility of the samples with the NIH‐3T3 cells was investigated by evaluating the cytotoxicity, cell morphology and hemolytic activity. The cell viability of NIH‐3T3 cells that are incubated with RPNPs (200 µg mL^−1^), Gen and RPNPs‐Gen was assessed by an MTT experiment. In the absence of light, the cell viability of all groups was above 91.3% compared with that of the control (Figure S9, Supporting Information). In addition, when the concentration of RPNPs increased to 800 µg mL^−1^, the cell viability was still above 80%, which suggests the great biocompatibility of RPNPs (Figure S10a, Supporting Information). The cell viability of RPNPs and RPNPs‐Gen slightly decreased but was still above 80% (*P* < 0.05, *n* = 3, vs control) under 808 nm irradiation for 30 min (**Figure** **4**a). However, with the irradiation time increased to 1 and 2 h, the cell viability of RPNPs‐Gen decreased to 40.8% and 11.2%, respectively, which implied that this irradiation time should not be too long to avoid a side effect, although long‐term irradiation could reduce the use of antibiotics (Figure S10b, Supporting Information). Therefore, we chose 30 min irradiation to perform photothermal treatment with the premise of high antibacterial performance and biosafety. From the result of the hemolytic assay (Figure 4b), all of the samples including the negative control (PBS) showed that the hemolysis percentage was less than 3% compared with that of the positive control (1% TritonX‐100).

**Figure 4:**
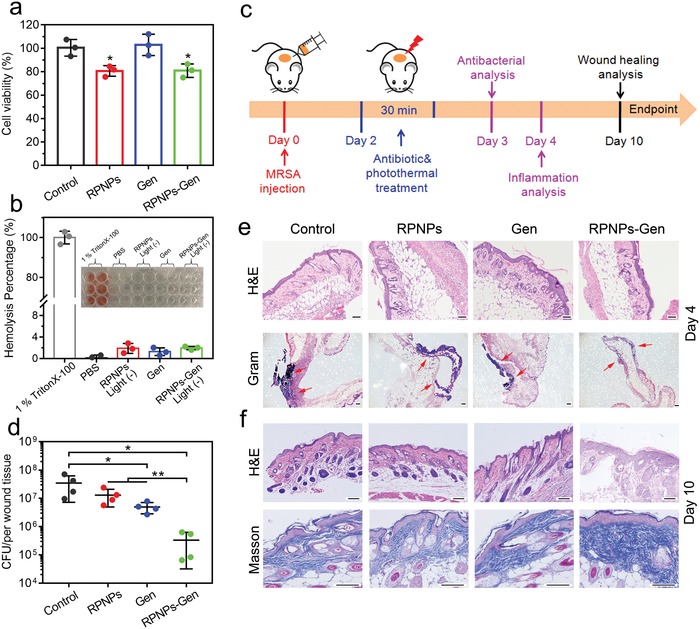
a) Cell viability of NIH‐3T3 cells under light was measured by MTT after one day coculture with samples. *n* = 3 independent experiments per group, *P < 0.05. b) Hemolysis percentage of samples and the corresponding images. c) Schematic of the combinatorial therapy protocol. d) CFU of MRSA in each wound tissue treated by control (PBS), RPNPs, Gen, and RPNPs‐Gen. *n* = 4 independent experiments per group, *P < 0.05, **P < 0.01. e) H&E and Gram‐staining images of wound tissues on day 4 (red arrows indicated MRSA). Scale bar, 100 µm. f) H&E and Masson staining images of wound tissues on day 10. Scale bar, 100 µm.

To further investigate the antibacterial behavior of combinatorial therapy in vivo, the mouse model of the MRSA wound infection was used. A schematic of the combinatorial therapy protocol is shown in Figure 4c. The wound infection model was created by cutting the back of each Balb/c mouse and injecting MRSA (2.0 × 10^7^) on Day 0. Since the existence of a high concentration of MRSA, the secretions or pus were found at the site of each wound, which suggests the infections occurred before the treatments were administered (Figure S11, Supporting Information). To investigate the residue number of MRSA on wounds after treatment, the wound tissues were separated to harvest MRSA and the CFU of the containing MRSA were enumerated with the spread plate method on day 3. As shown in Figure 4d, the untreated and infected controls had the highest number of viable MRSA (3.5 × 10^7^). There is no significant difference between the control and RPNPs (1.3 × 10^7^) groups, which suggests that only the photothermal treatment did not exhibit an obvious antibacterial effect. For the Gen group (5.0 × 10^6^), the viable MRSA only decreased within one order of magnitude due to the drug resistance of MRSA to Gen (*P* < 0.05, *n* = 4). When the combinatorial treatment (RPNPs‐Gen) was performed, the viable MRSA on the wounds (3.3 × 10^5^) significantly decreased 100‐fold compared with that of the control group (*P* < 0.05, *n* = 4). This result brought to light that, although MRSA showed resistance to Gen, the safe photothermal effect (45 °C) can still make the MRSA infection susceptible to the Gen treatment in vivo. This antibacterial performance of the combinatorial treatment in vivo was in line with the results in vitro.

To further evaluate the inflammation reaction of wounds, hematoxylin and eosin (H&E) and Gram staining were employed after 4 days. As shown in Figure 4e, the typical inflammation signs of the control, RPNPs and Gen groups were observed, which included numerous neutrophils, monocytes and lymphocytes. Moreover, many bacteria with a blue‐violet color stained by Gram staining were observed in these groups (indicated by red arrows). For a comparison, the group of RPNPs‐Gen showed a milder inflammation with fewer inflammatory cells, and the bacteria in the Gram stain were also reduced significantly. We can find that the wounds of mice were almost closed after being treated by RPNPs‐Gen, while the other three groups had still not recovered by day 10 (Figure S11, Supporting Information). The wound size measurement of each group also showed that the combinatorial treatment can accelerate wound closure in the healing process due to the elimination of infection (Figure S12, Supporting Information). H&E and Masson's trichrome staining were employed to evaluate the wound healing in a histomorphological aspect (Figure 4f). From the H&E results, many inflammatory cells were still found in the control and monotherapy groups after 10 days, whereas under combinatorial treatment, the inflammatory cells reduced substantially and a greater amount of granulation tissue was observed. The immunohistochemical staining showed that the expression of CD3 (the marker for lymphocytes) and MPO (the marker for neutrophils) were least in the RPNPs‐Gen group on day 4 or day 10, and the inflammatory cells decreased with time, which further proved the relief of inflammation reaction after combinatorial treatment (Figure S13, Supporting Information). In addition, the RPNPs‐Gen group showed the highest level of collagen Masson's trichrome staining compared with that of the other groups (Figure 4f; Figure S14, Supporting Information). Moreover, there is no sign of lesions in the H&E staining of liver, heart, spleen, lung, and kidney after 10 days (Figure S16, Supporting Information). Taken together, combining PTT and an inefficiency antibiotic can realize efficient antibacterial activity in vivo to treat MRSA infection and accelerate wound healing without causing side effects.

In summary, along with the discovery of new antibiotics, our study found a new exogenous strategy to resensitize multidrug‐resistant MRSA to aminoglycoside antibiotics without side effects, which would prolong the service life of old antibiotics. In the presence of the low‐temperature PTT effect that is produced by RPNPs and NIR, the inactivation behaviors of MRSA against aminoglycoside antibiotics were inhibited. The combination of low‐temperature PTT and inefficient aminoglycoside antibiotics exhibited great antibacterial performance against MRSA in vitro and in vivo without causing side effects. Although the animal model was only performed in the wound infection, other diseases, such as pneumonia, osteomyelitis, or implant‐associated MRSA infections, are also expected to be treated by applying additional thermal treatment when patients accept antibiotics therapy. The thermal effect can also be performed under a magnetic field, ultrasound or microwave to realize local heating into the deeper lesion. The concept of using exogenous modifying enzyme inhibitors to resensitize old antibiotics to MDR bacteria has the potential to alleviate the current stress of old antibiotic use and new antibiotic development.

## Conflict of Interest

The authors declare no conflict of interest.

## Supporting information

Supporting Information

Supplemental Table S1
