# Supplementary material for: Overcoming Multidrug‐Resistant MRSA Using Conventional Aminoglycoside Antibiotics
Source: Adv Sci (Weinh). 2020 Mar 14;7(9):1902070. doi: 10.1002/advs.201902070 (PMC7201259; doi:10.1002/advs.201902070)
Supplement: Supplementary file 1 — Supporting Information [file ADVS-7-1902070-s001.pdf]

Copyright WILEY-VCH Verlag GmbH & Co. KGaA, 69469 Weinheim, Germany, 2020.

ADVANCED SCIENCE
Open Access

Supporting Information

for *Adv. Sci.,* DOI: 10.1002/advs.201902070

Overcoming Multidrug-Resistant MRSA Using Conventional Aminoglycoside Antibiotics

Lei Tan, Ziao Zhou, Xiangmei Liu, Jun Li, Yufeng Zheng, Zhenduo Cui, Xianjin Yang, Yanqin Liang, Zhaoyang Li, Xiaobo Feng, Shengli Zhu, Kelvin Wai Kwok Yeung, Cao Yang, Xianbao Wang, and Shuilin Wu**

Supporting Information

Overcoming multidrug-resistant MRSA using conventional aminoglycosides antibiotics

Lei Tan, Ziao Zhou, Xiangmei Liu, Jun Li, Yufeng Zheng, Zhenduo Cui, Xianjin Yang, Yanqin Liang, Zhaoyang Li, Xiaobo Feng, Shengli Zhu, Kelvin Wai Kwok Yeung, Cao Yang, Xianbao Wang, Shuilin Wu**

1. Materials. $Zn(NO_3)_2{\bullet}6H_2O$, sodium dodecyl benzene sulfonate, NaOH, hydrochloric acid, ethanol, and RP were purchased from Sinopharm Chemical Reagent Co (China). ATP, ANS, Gen, Kan, Ami, Penicillin Pen, Rox and Ter were purchased from Aladdin Reagent Co. Sis was purchased from TCI Shanghai.

2. Preparation of RPNPs. Zn $(NO_3)_2{\bullet}6H_2O$ (1.785 g), NaOH (6 g) and sodium dodecyl benzene sulfonate (2.085 g) were dispersed in ethanol (60 mL) solution *via* sonication. The solution was then added to reaction kettle (100 mL) and reacted at 100 °C for 10 h. The solution was centrifuged at 9000 rpm for 10 min to remove the supernatant. The precipitate of ZnO nanoparticles were washed with deionized water and ethanol for three times, respectively, and then dried *via* lyophilization. To prepare RPNPs, the ZnO/RP was obtained through CVD. Briefly, the oxide layers of RP was removed through hydrothermal treatment at 200 °C for 12 h.[1] The pretreated RP (1.5 g) and ZnO (0.5 g) powders were mixed and grinded uniformly in a porcelain boat and then placed in a tube furnace. The furnace was kept in vacuum and the temperature was raised to 550 °C and kept for 15 min. Thereafter, the temperature was decreased to 280 °C and kept for 10 h. After cooling, to remove the ZnO in

ZnO/RP, the dark gray powder was dispersed in hydrochloric acid solution and stirred for 2 h. The black precipitation was washed with deionized water for 3 times and dried *via* lyophilization. Before using, the powder was dispersed in DI H_2O and treated with an ultrasonic homogenizer (JY92-ⅡN, Ningbo Scientz Biotechnology Co. China) for 30 min at the power of 400 W.

3. Characterization. TEM and EDS images were obtained from a high resolution transmission electron microscope (HRTEM, Talos F200S). The morphology of MRSA was observed by FE-SEM (sigma 500, Germany). Raman spectra were collected from a Confocal Raman Microspectroscopy (Renishaw, UK). The UV-vis spectra of RP and RPNPs were collected from a microplate reader (SpectraMax i3, Molecular Devices). The fluorescence images of NIH3T3 cells were obtained from an Inverted Fluorescence Microscope (IFM, Olympus, IX73).

4. Photothermal properties measurements. Different concentrations of RPNPs solution (100, 200 and 400 μg/mL) and RP (200 μg/mL) were irradiated under 808 nm laser (1 W cm^{-2}) for 10 min, respectively. The heating temperature of samples was measured through the infrared thermal camera (FLIR E50). The photothermal conversion efficiency of RPNPs was calculated following Lu report.[2]

5. *In vitro* antibacterial assay. MRSA (CCTCC AB 2015108) was obtained from China Center for Type Culture Collection. The MRSA were cultured in the standard Luria-Bertani (LB) culture medium. Gen, Kan, Ami, Sis, Pen, Rox and Tet were chosen to combine with RPNPs. According to the standard method,[3] the MIC of Gen, Kan, Ami, Sis, Pen, Rox and Tet were measured to be 256, 512, 256, 8192, 16,

512 and 16 μg/mL, respectively. For the Gen, different concentration of 1×MIC, 4×MIC, 16×MIC and 32×MIC were used to evaluate the growth inhibition when combined with photothermal treatment. The experiment groups could be divided as control (no treatment), RPNPs (200 μg/mL), Gen and RPNPs-Gen (200 μg/mL, 1×MIC). 150 μL of bacterial suspension (5×10^6 CFU/mL) was added to each group and subsequently irradiated with 808 nm laser (0.5 W cm^{-2}, Hi-Tech Optoelectronics Co., Lid, China) for 30 min at 45 °C. 20 μL diluted bacterial solution of each sample was spread on solid LB agar plates every 2 h until 8 hours and cultured at 37 °C for 24 h. The colony-forming units were counted to evaluate the growth state of MRSA in different groups. The MIC was also measured as above after combining with photothermal treatment. For the experiments of Kan, Ami, Sis, Rox, Pen, and Tet, the used concentration of these antibiotics were 1×MIC, 2×MIC, 0.125×MIC, 0.25×MIC, 0.5×MIC and 0.5×MIC, respectively.

6. *In vitro* antibiofilm assay. MRSA were cultured in the culture medium for 48 h to form biofilm on 96-well plate, and the culture medium was changed every 24 h. The experiment groups were divided into control (no treatment), RPNPs (200 μg/mL), Gen (20×MIC) and RPNPs-Gen (200 μg/mL, 20×MIC). After treatment as above, the biofilms were fixed by glutaraldehyde for 2 h and then dehydrated with gradient ethanol solutions before SEM observation. To investigate the live/dead state of MRSA, the MRSA biofilm were stained by a LIVE/DEAD BackLight bacterial viability kit (Invitrogen).

7. Bacterial membrane permeability assay. ANS was used to investigate the membrane permeability change after treatments.[4] The experiment groups could be divided as control (no treatment), 1% TritonX-100, RPNPs (200 μg/mL), Gen (4×MIC) and RPNPs-Gen (200 μg/mL, 4×MIC). The MRSA was washed with PBS and resuspended in PBS solution (10^8 CFU/mL). ANS (10 μM) was added to the washed MRSA and equilibrated for 30 min. After 30 min treatments as above, the fluorescence emission of samples was detected under excitation at 380 nm through microplate reader. Then, the leakage of protein from MRSA (10^7 CFU/mL) of each group was also measured by a BCA kit (Beyotime, China). After treatments, the MRSA solution was centrifuged at 5000 rpm/min for 10 min at 3 °C. The supernatant (40 μL) was taken out and added to BCA working solution (200 μL). After 30 min incubation at 37 °C, the optical density (OD) value of each group was measured at 562 nm.

8. Proteomics evaluation. The MRSA treated by Gen and RPNPs-Gen were first lysed with SDT solution (4% Sodium dodecyl sulfate, 100 mM Tris-HCl, 1 mM dithiothreitol, pH 7.6) and heated with boiling water for 15 min. after centrifugation at 14000 g for 30 min, the supernatant were obtained and then quantified by BCA kit. The protein enzymolysis process was operated FASP (Filter-aided sample preparation) method.[5] The peptides of samples were labeled with TMT reagent (Thermo Scientific). The TMT-labeled peptides were fractionated using a fractionation kit (Thermo Scientific). Then, the samples were fractionated by HPLC (high performance liquid chromatography) system (Easy nLC, Thermo Scientific) in

Shanghai Applied Protein Technology Co., Ltd. After that, the samples were analyzed by a mass spectrometry (Q Exactive, Thermo Scientific). For GO annotation, the protein sequences were compared with Staphylococcus aureus subsp. aureus NCTC 8325 database. For KEGG pathway annotation, the KEGG Automatic Annotation Server software was used to perform KEGG annotation. The differential proteins were compared with the online KEGG database (http://www.kegg.jp/) to extract the corresponding KEGG pathway. The enrichment analysis of GO and KEGG annotation were performed using Fisher’s Exact Test. The PPI information of differential proteins were obtained from IntAct molecular interaction database (http://www.ebi.ac.uk/intact/) using their corresponding gene symbols. The PPI network was created by Cytoscape software.

9. Enzyme activity detection of APH (2"). Based on the accession of APH (2") (AND45417.1) from the result of proteomics evaluation, the APH (2") (77 kDa) was provided by Servicebio Technology CO., LTD through constructing vector of pET32a-APH, transforming into the Escherichia coli strain BL21 (DE3) and protein purification. The expressed and purified APH (2") was shown in Figure S15 (Supporting Information). Next, ATP (20 μL, 10 mM), Gen (20 μL, 1 mg/mL) and APH (2") (90 μL, 1.5 mg/mL) or DI water (90 μL) were added to HEPES (100 μL, pH 7.5) solution and placed at 37 °C and 48 °C for 30 min, respectively. Subsequently, the samples were centrifuged at 12000 g for 5 min and the ATP in the supernatant was determined by ATP assay kit. The fluorescence intensity of ATP was recorded by microplate reader. The consumption of ATP was calculated from the

standard curve of ATP. The generation of Gen-P was calculated from the consumption of ATP over time.

10. Molecular dynamics simulation. The MD simulation was carried out by GROMACS 5.1.4 to analyze the conformation change of APH (2") and examine the interaction between APH (2") and Gen at 37 °C and 48 °C (100 ns), respectively. The system was optimized by steepest descent method to realize energy minimization. The protein was solvated into water using SPC model and placed into a cubic box. Na^+ and Cl^- were added to neutralize the system. The protein modeling was performed with Modeller 9.20 and we chose high similarity protein template (4ORK, 4OCJ, 3TDV, 3N4V, 3HAM, 3ATT, 2QG7, 2QG7 and 1ZYL) through blast (https://blast.ncbi.nlm.nih.gov/Blast.cgi) to model.

11. *In vitro* cytotoxicity evaluation. NIH-3T3 (mouse embryonic fibroblast cell line) cells were cultured in MEM/EBSS medium (HyClone) and with 10% (v/v) fetal bovine serum and 1% penicillin-streptomycin solution (HyClone) at 37 °C in an atmosphere with 5% CO_2 and 95% humidity. The culture medium was changed every 3 days. MTT assay was used to study the cytotoxicity of samples towards the NIH-3T3. The experiment groups could be divided as control (no treatment), RPNPs (200 μg/mL), Gen (0.5×MIC) and RPNPs-Gen (200 μg/mL, 0.5×MIC). NIH-3T3 cells were incubated in 96-well plate for 24 h before experiment. Then the above samples were dispersed in culture medium and added to each well. After incubation for 1 day, the culture mediums were replaced by MTT (0.5 mg/mL, 200 μL, Aladdin Reagent Co., China) solution. After incubating with MTT solution for 4 h, the

solution was replaced by DMSO (200 μL). The absorption at 570 nm was measured through a microplate reader (SpectraMax I3MD USA). The MTT experiment for the groups of RPNPs-Gen (200 μg/mL, 0.5×MIC) was also investigated when irradiated with 808 nm laser for different time. The cell viability was calculated from the optical density (OD) value.

12. Hemolytic measurement. The hemolytic performance of samples was tested using New Zealand rabbit blood. 5 mL blood was diluted with 50 mL PBS solution and centrifuged at 1000 g for 6 min to obtain red blood cells. The red blood cells were washed with PBS for 3 times and resuspended in 20 mL PBS solution. 5 groups of PBS (negative control), 1% TritonX-100 (positive control), RPNPs, Gen and RPNPs-Gen were mixed with red blood cells solution at the same concentration referred to MTT experiment. After 4 h incubation, the solutions were centrifuged at 1000 g for 6 min and the supernatant were taken out to a 96-well plate. Subsequently, the OD values of supernatant were measured at 451 and 405 nm.

13. Mouse infection model. All animal experiments were approved by the animal research committee of Tongji Medical College, Huazhong University of Science and Technology (Wuhan, China). 8-week-old male Balb/c mice were purchased from the Hubei Provincial Centers for Disease Prevention & Control. The experiment groups could be divided as control (PBS, 20 μL), RPNPs (4 mg/mL, 20 μL), Gen (10 mg/mL, 20 μL) and RPNPs-Gen (4 mg/mL, 20 μL; 10 mg/mL, 20 μL). Before surgery, the mice were anaesthetized through intraperitoneal injection using 3% pentobarbital. The back of mice were shaved and cut using a biopsy punch. Subsequently, 2.0×10^{7} CFU

MRSA was injected into the wound of each mouse. These wounds were then covered by bandage. After 2 days infection, the samples were added and following by carrying out 808 nm laser treatment (0.3 W cm^{-2}) for 30 min at 45 °C. The wound tissues were cut to harvest MRSA and the CFU of the containing MRSA were calculated through spread plate method on day 3. On day 4, the wound tissues were cut, fixed by formalin, dehydrated, paraffined and sectioned. The Hematoxylin-Eosin (H&E) and Gram staining was used to analyze the inflammatory reaction and residue bacteria of the wounds. On Day 10, the condition of wound healing was investigated by Hematoxylin-Eosin (H&E) and Masson staining. The wounds were observed and photographed on day 0, 2, 4, 8 and 10, respectively. The histological analysis of heart, liver, spleen, lung and kidney were also performed *via* H&E staining after 10 days. The collagen content from Masson staining and wounds areas (%) were calculated by Image J software.

14. Statistical analysis. All experiments were shown as mean values ± standard deviation of at least three tests. A one-way analysis of variance (ANOVA) program combined with a student t-test was used to evaluate the statistical significance of the variance and a difference of *$P < 0.05$, **$P < 0.01$ and ***$P < 0.001$ were considered significant.

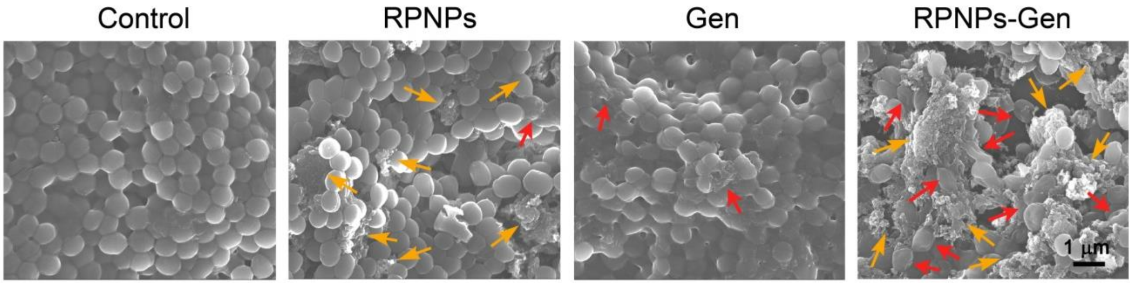

Figure S1. a) SEM images (yellow arrow indicates RPNPs and red arrow indicates the damaged cell membranes) of MRSA biofilm shows that the MRSA membranes in the groups of the control, RPNPs and Gen were almost typically spherical and exhibited no obvious change, suggesting that the antibiotic and PTT treatments did not influence the survival of MRSA. However, in the group of RPNPs-Gen, the cell membranes were shrinking and lost their integrity.

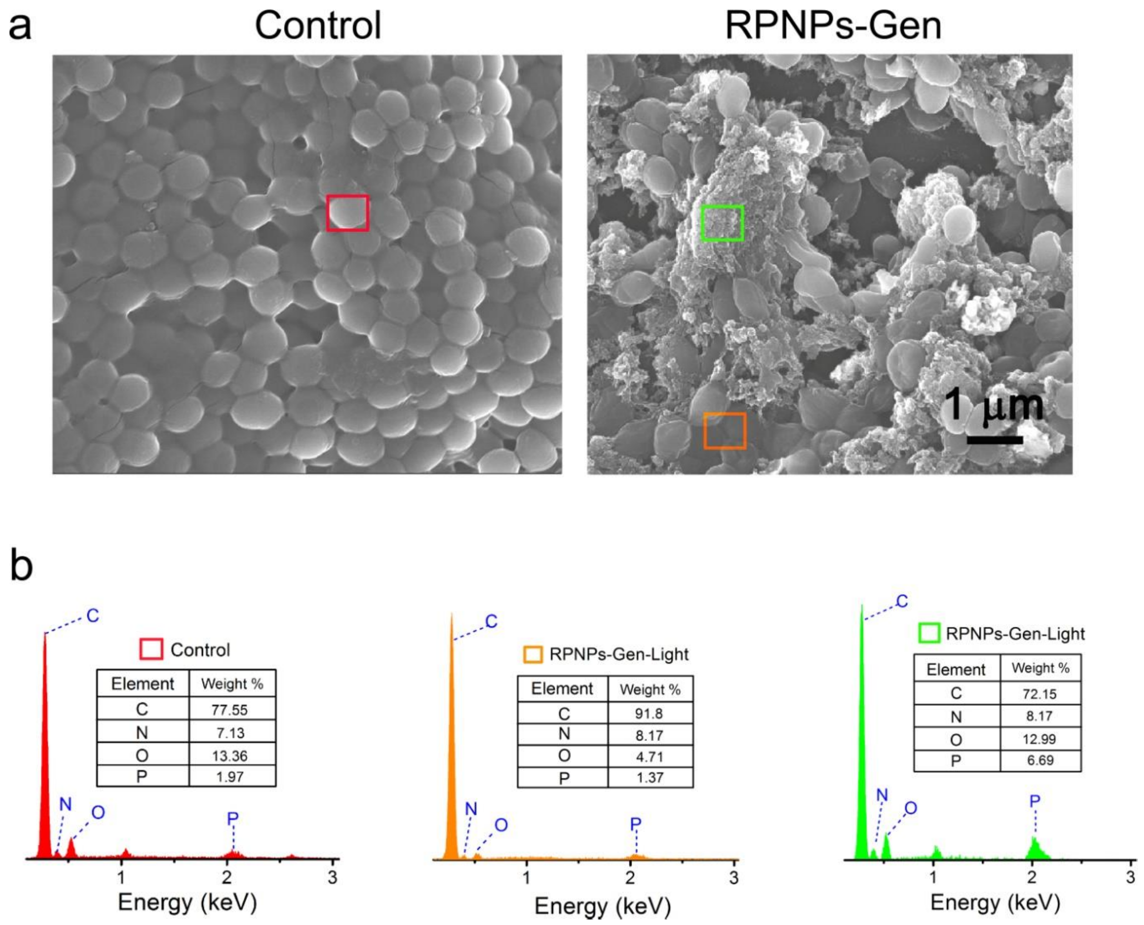

Figure S2. a,b) SEM and EDS images of selected area in the control and RPNPs groups shows that some particles were observed in the groups of RPNPs and RPNPs-Gen (indicated by the yellow arrow), which were proven to be RPNPs due to the increased content of the P element and which suggests that the nanoparticles could permeate into biofilm and benefit the antibiotic treatment.

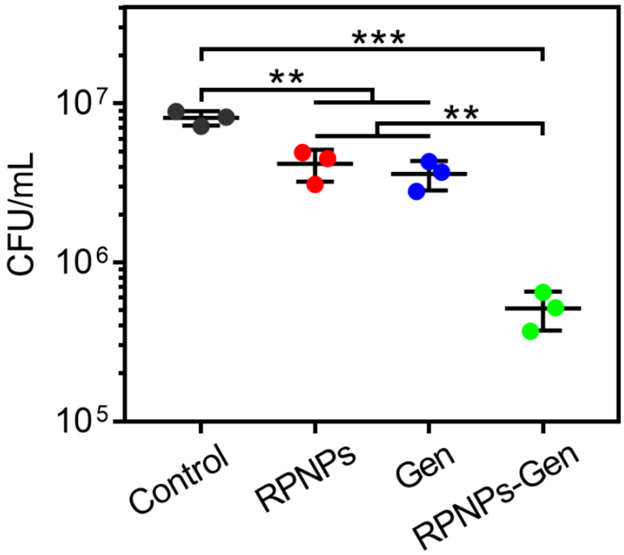

Figure S3. CFU of biofilm in the groups of Control, RPNPs, Gen and RPNPs-Gen shows that the number of MRSA in the RPNPs-Gen was obviously decreased compared with that of other groups. n = 3 independent experiments per group, $^{**}P < 0.01$, $^{***}P < 0.001$.

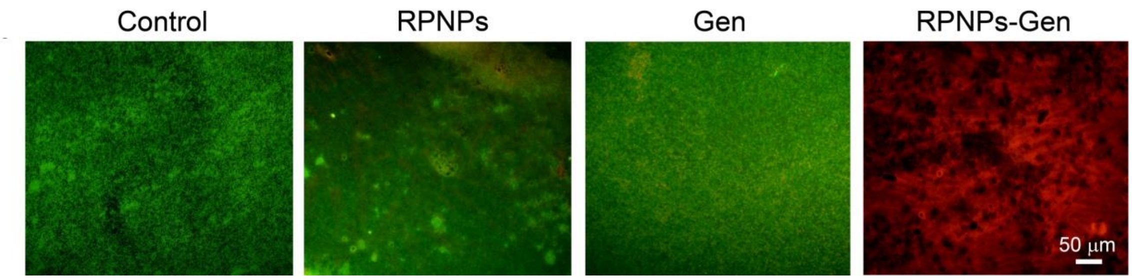

Figure S4. Live/dead staining images of MRSA biofilm shows that there were more dead bacteria (red color) in the single treatment than live bacteria (green) in the control group. However, overall, most of the bacteria were alive. In contrast, widespread red fluorescence was exhibited after the combinatory treatment. This result suggested that MRSA biofilm could also be eradicated due to the size of the RPNPs and the potentiation of the PTT effect.

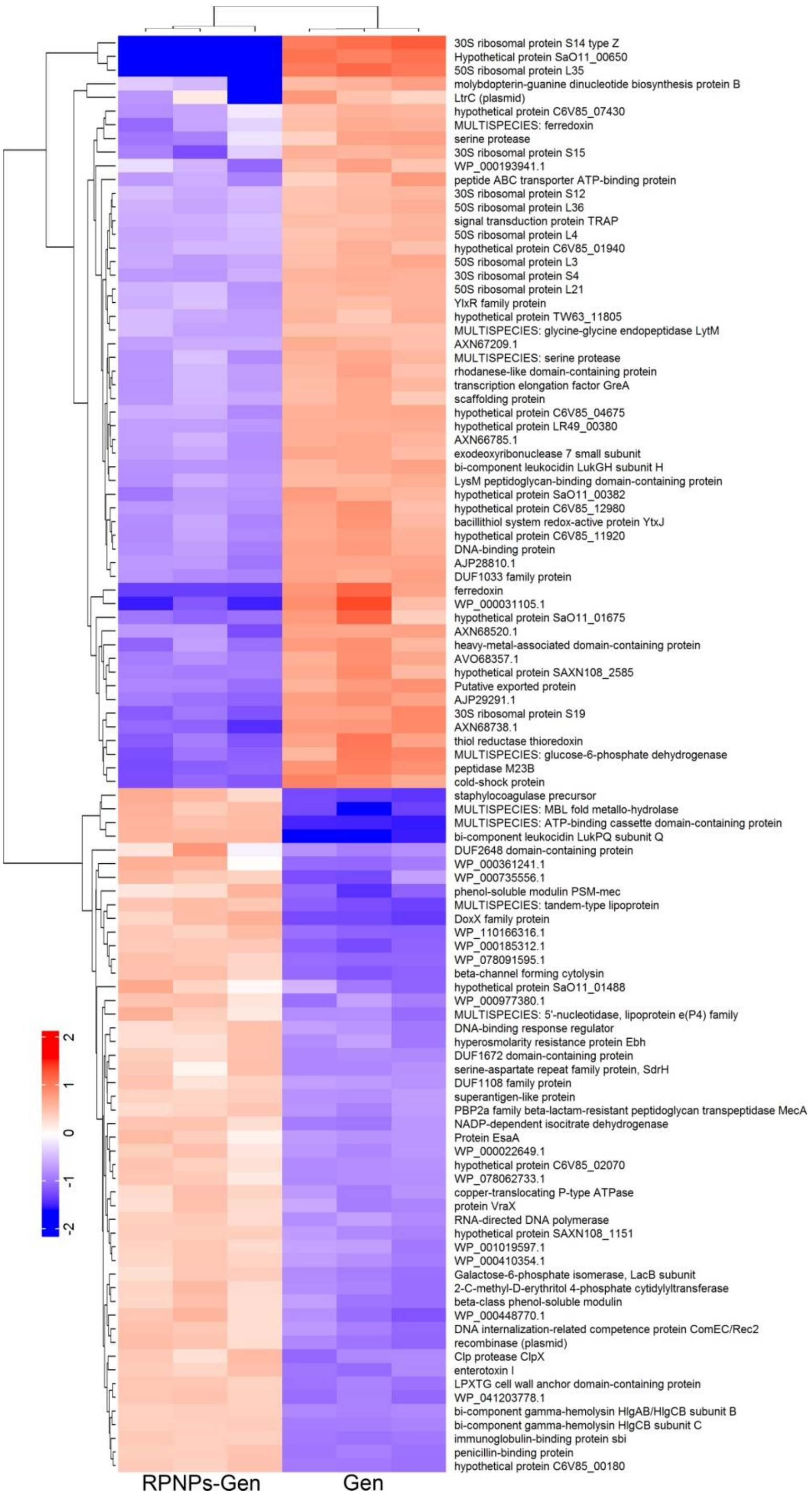

Figure S5. Heat map shows the upregulated and downregulated proteins of MRSA treated by Gen and RPNPs-Gen (RPNPs-Gen vs Gen, fold change > 2, $P < 0.05$).

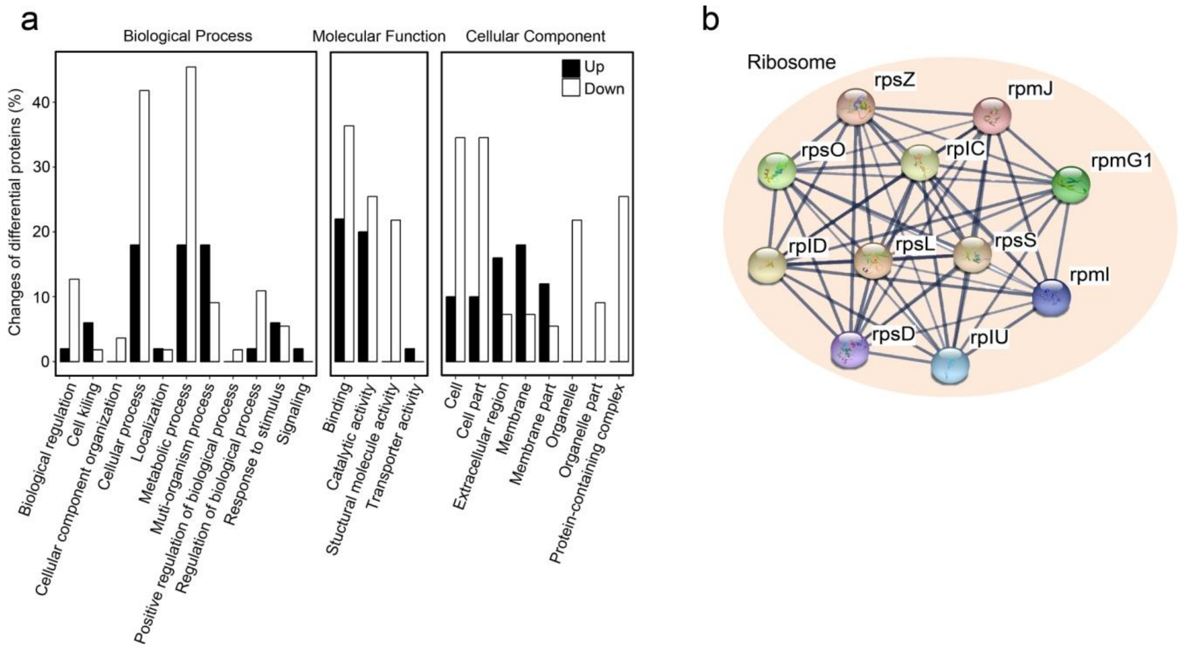

Figure S6. a) The Gene Ontology analysis of differential proteins. b) The protein-protein interaction (PPI) result shows that all of the 11 ribosomal proteins had strong interaction and belonged to the ribosomal pathway.

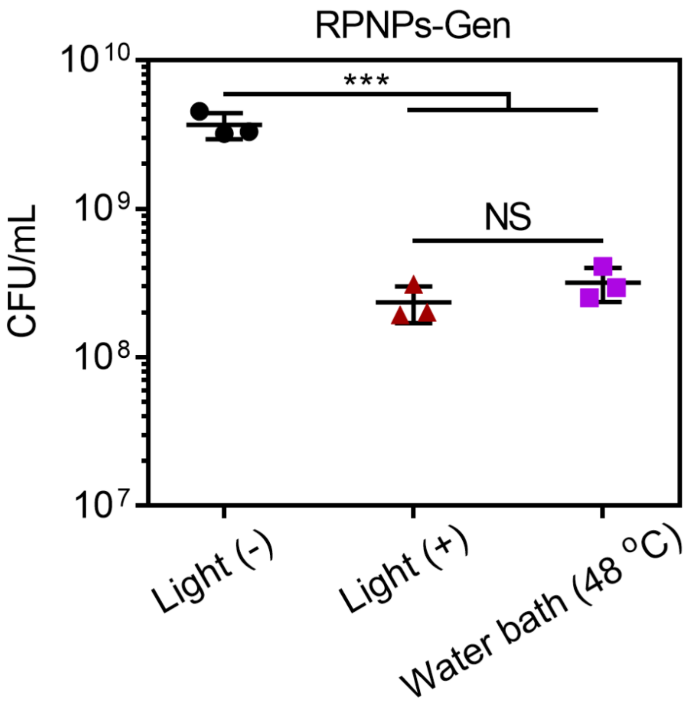

Figure S7. Antibacterial performance of RPNPs-Gen under the water bath (48 °C) condition compared with NIR irradiation. n = 3 independent experiments per group, $^{***}P < 0.001$.

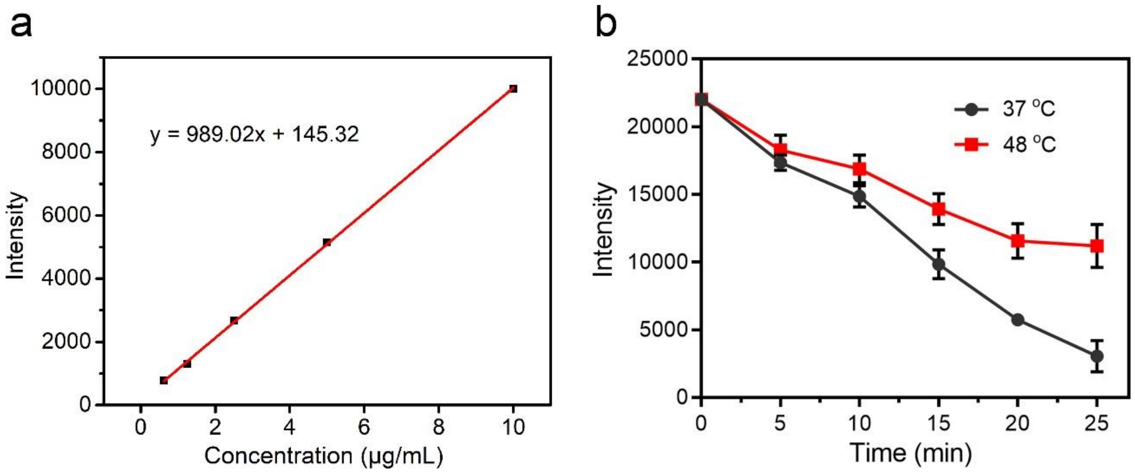

Figure S8. a) Standard curve of ATP. b) The detection of ATP at different temperature over time.

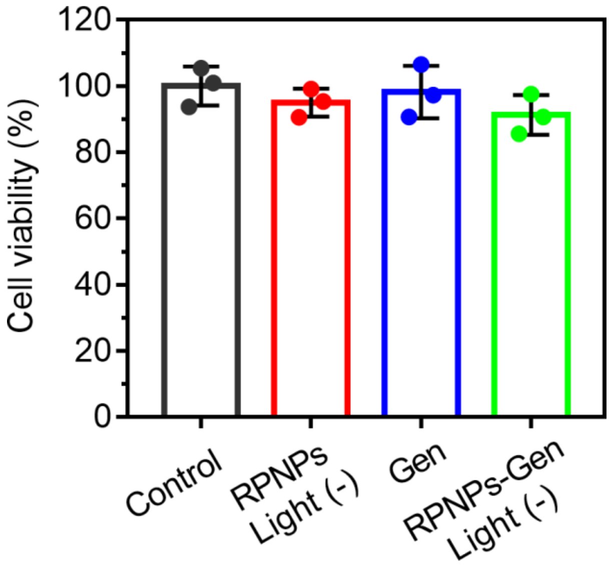

Figure S9. Cell viability of NIH-3T3 cells without light was measured by MTT after 1 day's coculture with samples.

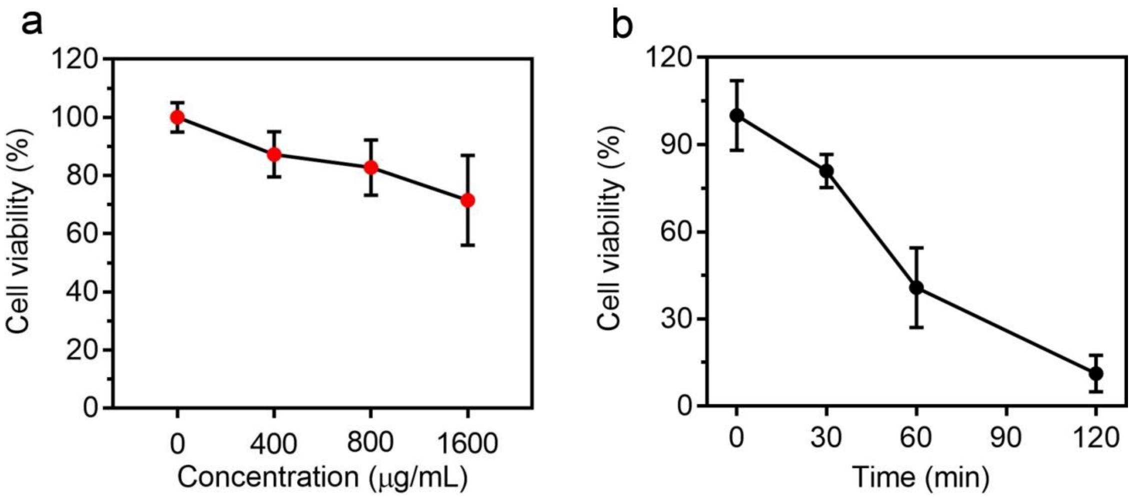

Figure S10. a) Cell viability of NIH-3T3 treated by RPNPs at different concentrations after 1 day. n = 3 independent experiments per group. b) Cell viability of NIH-3T3 cells treated by RPNPs-Gen with the irradiation time increased.

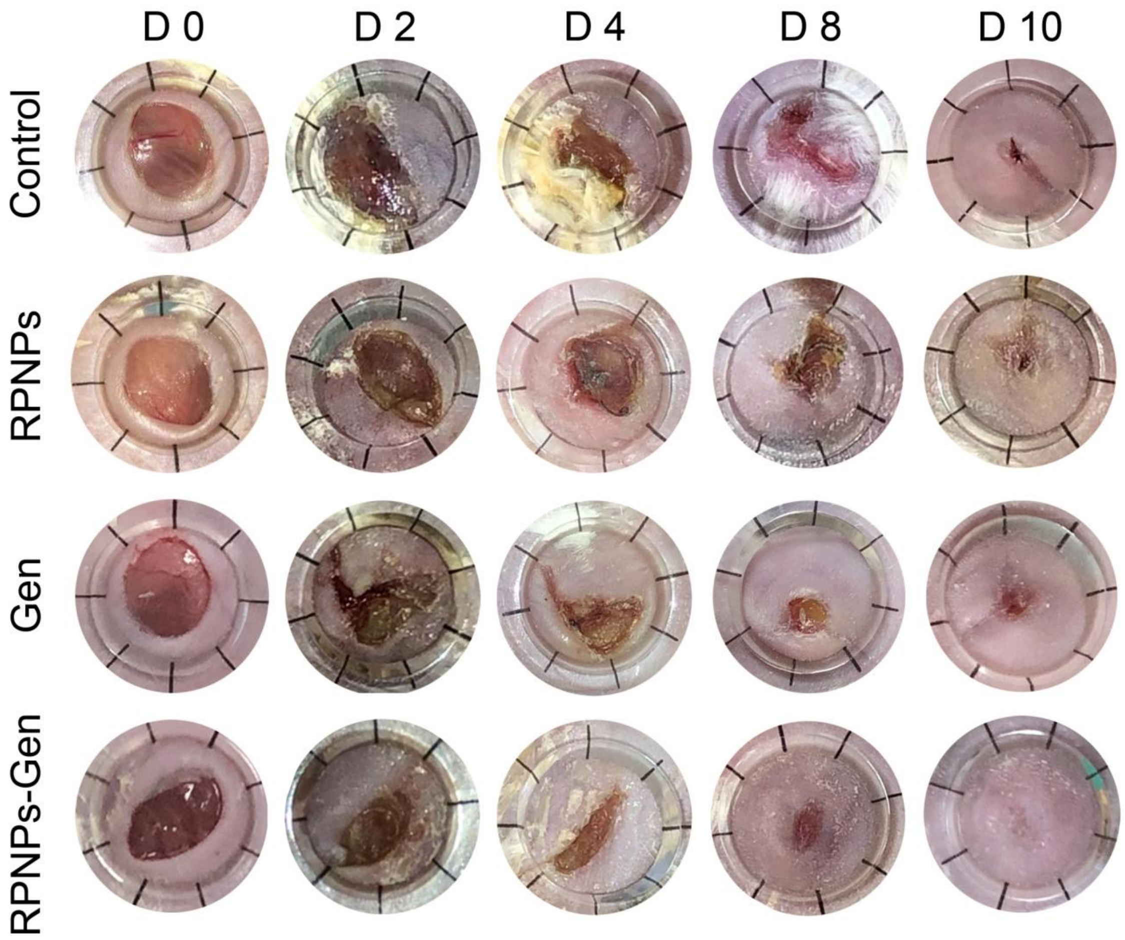

Figure S11. Wound photographs of rats at day 0, 2, 4, 8 and 10. The diameter of ruler is 1 cm.

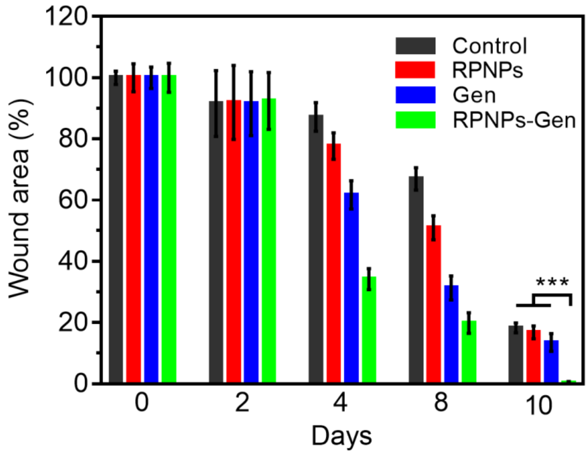

Figure S12. Wound size measurement of each group in the healing process. n = 4 independent experiments per group, ****P* < 0.001.

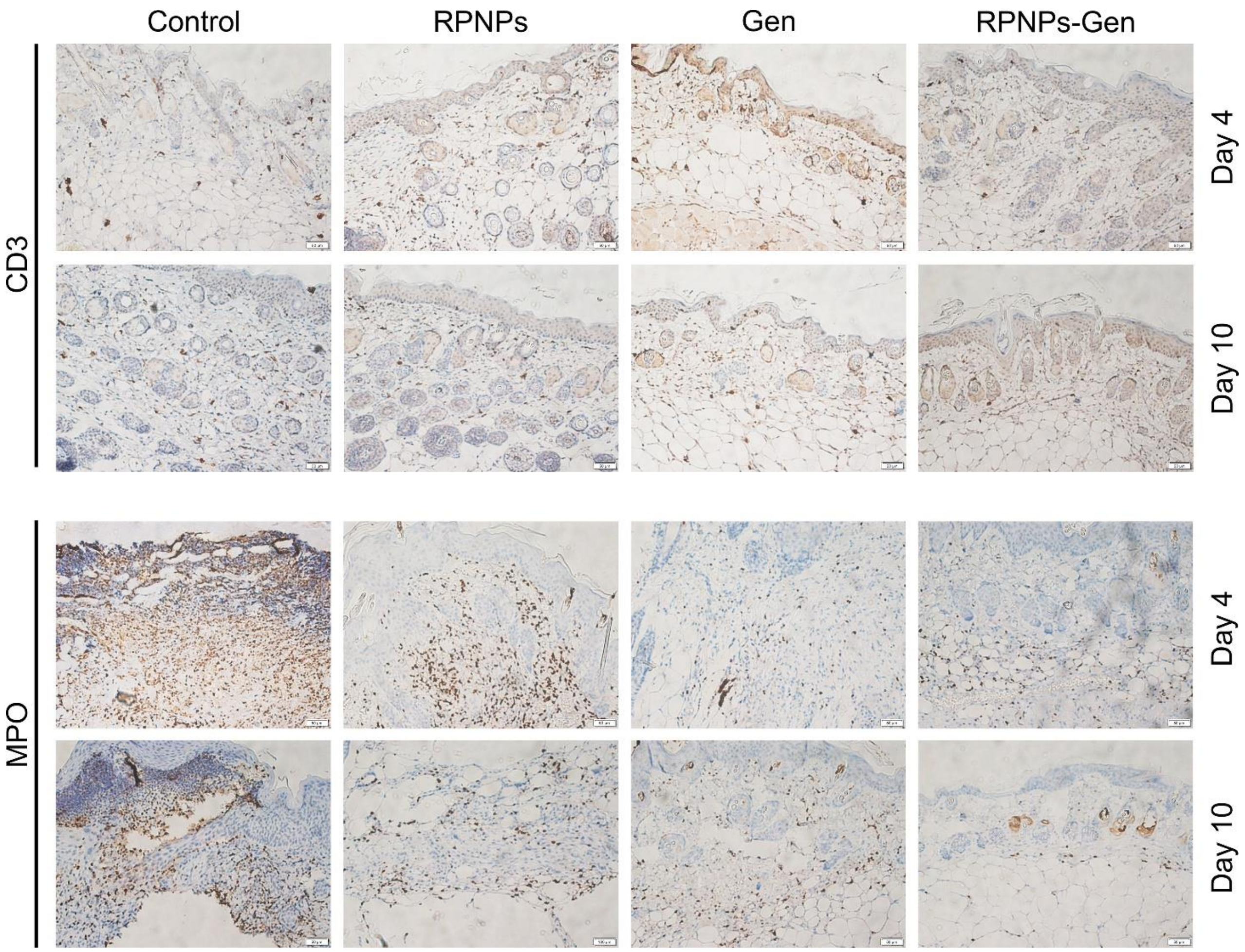

Figure S13. Immunohistochemical staining of CD3 for lymphocytes and MPO for neutrophils in wound tissues (scale bars, 50 μm).

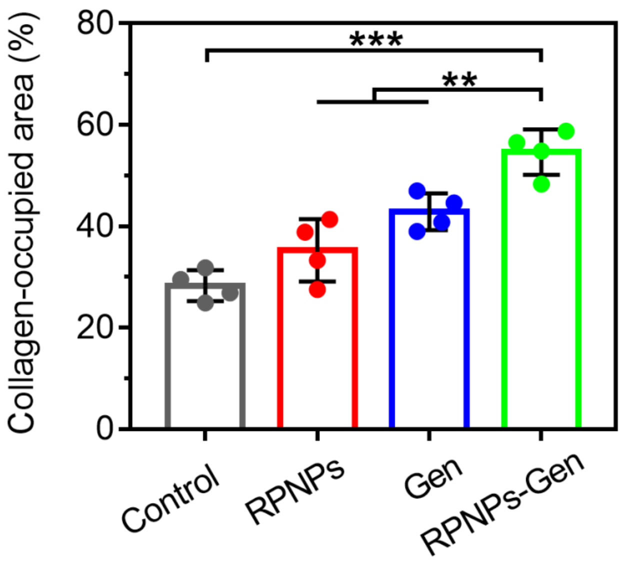

Figure S14. Level of collagen in the wound tissue of each group on day 10. n = 4 independent experiments per group, $**P < 0.01$, $***P < 0.001$.

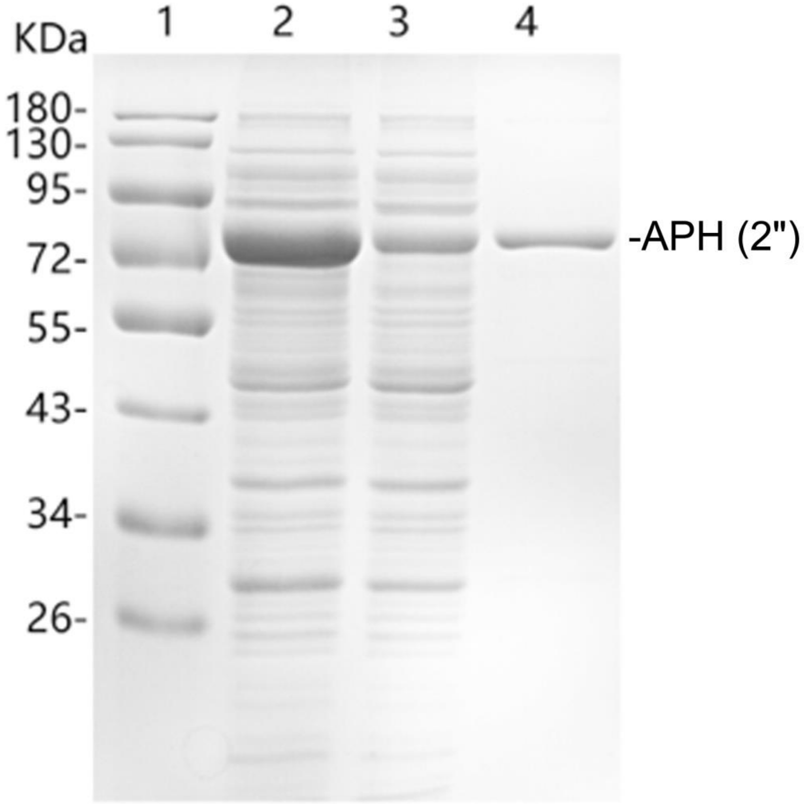

Figure S15. Image of expressed and purified APH (2") from *Escherichia coli* strain BL21 (DE3) (1: marker; 2: supernatant of bacteria after lysis; 3: supernatant after loading; 4: eluted protein from column.

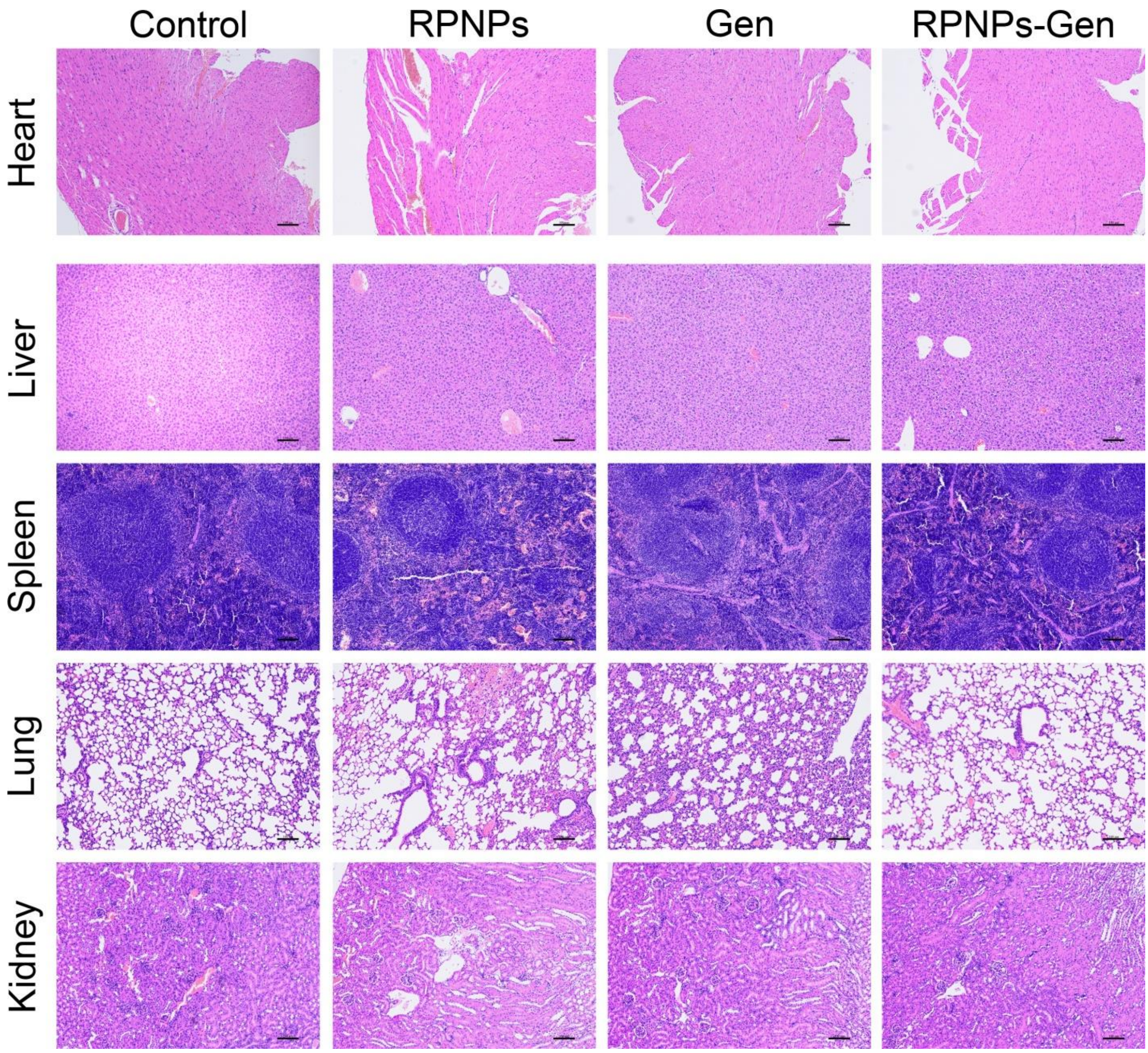

Figure S16. H&E staining of liver, heart, spleen, lung, and kidney on day 10 (scale bar = 100 μm).

References

[1] L. Tan, J. Li, X. Liu, Z. Cui, X. Yang, S. Zhu, Z. Li, X. Yuan, Y. Zheng, K. W. K. Yeung, H. Pan, X. Wang, S. Wu, *Adv. Mater.* **2018**, *30*, 1801808.

[2] N. Lu, P. Huang, W. Fan, Z. Wang, Y. Liu, S. Wang, G. Zhang, J, Hu, W. Liu, G. Liu, R. D. Leapman, G. Lu, X. Chen, *Biomaterials* **2017**, *126*, 39.

[3] J. X. Huang, M. A. T. Blaskovich, R. Pelingon. S. Ramu, A. Kavanagh, A. G. Elliott, M. S. Butler, A. B. Montgomery, M. A. Cooper, *Antimicrob. Agents Chemother.* **2015**, *59*, 5925.

[4] L. Schnaider, S. Brahmachari, N. W. Schmidt, B. Mensa, S. Shaham-Niv, D. Bychenko, L. Adler-Abramovich, L. J. W. Shimon, S. Kolusheva, W. F. DeGrado & E. Gazit. *Nat. Commun*. **2017**, *8*, 1365.

[5] J. R. Wisniewski, A. Zougman, N. Nagaraj & M. Mann, *Nat. Methods*, **2009**, *6*, 359.
